# Supplementary material for: Baseline clinical features of COVID-19 patients, delay of hospital admission and clinical outcome: A complex relationship
Source: PLoS One. 2022 Jan 7;17(1):e0261428. doi: 10.1371/journal.pone.0261428 (PMC8741026; doi:10.1371/journal.pone.0261428)
Supplement: S1 Fig — (DOCX) [file pone.0261428.s001.docx]

**S1 Fig. Distribution of the variable “Delay between symptom onset and hospital admission”**

The parameters of the distribution are the following: number of included patients n=827, median delay between symptom onset and hospital admission = 5 days, Q1=3 days, Q3=9 days, mean delay between symptom onset and hospital admission = 6.06 days, standard deviation = 4.56 days, Shapiro-Wilk test for normality: p<0.01.
