# Supplementary material for: Baseline clinical features of COVID-19 patients, delay of hospital admission and clinical outcome: A complex relationship
Source: PLoS One. 2022 Jan 7;17(1):e0261428. doi: 10.1371/journal.pone.0261428 (PMC8741026; doi:10.1371/journal.pone.0261428)
Supplement: S1 Table — (DOCX) [file pone.0261428.s002.docx]

|  |  | **Delay between symptom onset and hospital admission (days)** | | | |  |
| --- | --- | --- | --- | --- | --- | --- |
| **Characteristics** | **All patients  n= 827** | **Symptom onset between the day of hospital admission and 3 days (included) before hospital admission n=278** | **Symptom onset between 4 and 5 days (included) before hospital admission  n=143** | **Symptom onset between 6 and 9 days (included) before hospital admission  n=235** | **Symptom onset 10 days or more before hospital admission n=171** | **p-value** |
| Male gender, n (%) | 462 (55.9) | 144 (51.8) | 75 (52.5) | 139 (59.2) | 104 (60.8) | 0.15 |
| Age, median (IQR) | 73 (61-84) | 82 (67-88) | 74 (61-85) | 69 (52-78) | 68 (58-78) | <0.01 |
| Age ≥ 75 y., n (%) | 384 (46.4) | 178 (64.0) | 68 (47.6) | 83 (35.3) | 55 (32.2) | <0.01 |
| BMI, median (IQR) | 26.2 (23.1-29.7) [664] | 26.2 (22.6-29.7) [225] | 25.2 (22.9-28.6) [124] | 26.9 (23.6-30.0) [183] | 26.5 (23.8-29.8) [132] | 0.04 |
| BMI ≥ 30, n (%) | 153 (23.0) [664] | 54 (24.0) [225] | 22 (17.7) [124] | 45 (24.6) [183] | 32 (24.2) [132] | 0.49 |
| Comorbidities, n (%) |  |  |  |  |  |  |
| *Cardiovascular disease* | 440 (53.2) | 184 (66.2) | 72 (50.4) | 107 (45.5) | 77 (45.0) | <0.01 |
| *Diabetes* | 191 (23.1) | 73 (26.3) | 33 (23.1) | 45 (19.2) | 40 (23.4) | 0.30 |
| *Malignancy* | 146 (17.7) | 67 (24.1) | 25 (17.5) | 25 (10.6) | 29 (17.0) | <0.01 |
| *Chronic kidney disease* | 115 (13.9) | 57 (20.5) | 15 (10.5) | 16 (6.8) | 27 (15.8) | <0.01 |
| *Chronic lung disease* | 103 (12.5) | 40 (14.4) | 20 (14.0) | 31 (13.2) | 12 (7.0) | 0.11 |
| *Chronic liver disease* | 55 (7.5) [736] | 18 (6.8) | 10 (7.7) | 14 (7.3) | 13 (8.8) | 0.90 |
| *Immunodeficiency* | 48 (5.8) | 17 (6.1) | 10 (7.0) | 10 (4.3) | 11 (6.4) | 0.67 |
| Smoking status, n (%) | [626] | [198] | [106] | [175] | [147] | 0.64 |
| *Current smoker* | 29 (4.6) | 10 (5.1) | 8 (7.6) | 6 (3.4) | 5 (3.4) |  |
| *Ex-smoker* | 197 (31.5) | 62 (31.3) | 35 (33.0) | 51 (29.1) | 49 (33.3) |  |
| *Never smoker* | 400 (63.9) | 126 (63.6) | 63 (59.4) | 118 (67.4) | 93 (63.3) |  |

|  |  | **Delay between symptom onset and hospital admission (days)** | | | |  |
| --- | --- | --- | --- | --- | --- | --- |
| **Characteristics** | **All patients  n= 827** | **Symptom onset between the day of hospital admission and 3 days (included) before hospital admission n=278** | **Symptom onset between 4 and 5 days (included) before hospital admission  n=143** | **Symptom onset between 6 and 9 days (included) before hospital admission  n=235** | **Symptom onset 10 days or more before hospital admission n=171** | **p-value** |
| Temperature at admission, median (IQR) | 38.0 (37.1-38.6) [739] | 37.9 (37.0-38.4) [255] | 38.0 (37.5-38.6) [128] | 38.0 (37.0-38.8) [207] | 38.0 (37.2-38.6) [149] | 0.06 |
| Symptoms at admission, n (%) |  |  |  |  |  |  |
| *History of fever/chills* | 674 (81.5) | 207 (74.5) | 123 (86.0) | 205 (87.2) | 139 (81.3) | <0.01 |
| *Weakness* | 584 (70.6) | 177 (63.7) | 105 (73.4) | 170 (72.3) | 132 (77.2) | 0.01 |
| *Cough* | 560 (67.7) | 147 (52.9) | 105 (73.4) | 183 (77.9) | 125 (73.1) | <0.01 |
| *Shortness of breath* | 550 (66.5) | 167 (60.1) | 90 (62.9) | 165 (70.2) | 128 (74.9) | <0.01 |
| *Diarrhea* | 229 (27.8) | 60 (21.6) | 43 (30.1) | 75 (31.9) | 51 (29.8) | 0.05 |
| *Pain* | 225 (27.2) | 64 (23.0) | 33 (23.1) | 77 (32.8) | 51 (29.8) | 0.05 |
| *Myalgia* | 144 (17.4) | 37 (13.3) | 23 (16.1) | 55 (23.4) | 29 (17.0) | 0.03 |
| *Abdominal pain* | 62 (7.5) | 23 (8.3) | 13 (9.1) | 14 (6.0) | 12 (7.0) | 0.04 |
| *Chest pain* | 50 (6.0) | 11 (4.0) | 8 (5.6) | 16 (6.8) | 15 (8.8) | 0.45 |
| *Joint pain* | 12 (1.5) | 4 (1.4) | 1 (0.7) | 4 (1.7) | 3 (0.2) | 0.92 |
| *Nausea* | 107 (12.9) | 30 (10.8) | 20 (14.0) | 38 (16.2) | 19 (11.1) | 0.27 |
| *Headache* | 105 (12.7) | 29 (10.4) | 14 (9.8) | 41 (17.5) | 21 (12.3) | 0.07 |
| *Confusion* | 93 (11.3) | 48 (17.3) | 15 (10.5) | 19 (8.1) | 11 (6.4) | <0.01 |
| *Runny nose* | 72 (8.7) | 17 (6.1) | 16 (11.2) | 20 (8.5) | 19 (11.1) | 0.19 |
| *Ageusia* | 64 (7.7) | 7 (2.5) | 7 (4.9) | 29 (12.3) | 21 (12.3) | <0.01 |

|  |  | **Delay between symptom onset and hospital admission (days)** | | | |  |
| --- | --- | --- | --- | --- | --- | --- |
| **Characteristics** | **All patients  n= 827** | **Symptom onset between the day of hospital admission and 3 days (included) before hospital admission n=278** | **Symptom onset between 4 and 5 days (included) before hospital admission  n=143** | **Symptom onset between 6 and 9 days (included) before hospital admission  n=235** | **Symptom onset 10 days or more before hospital admission n=171** | **p-value** |
| Temperature at admission, median (IQR) |  |  |  |  |  |  |
| White blood cells (G/L) | 6.36 (4.87-8.59) [769] | 6.42 (4.88-8.83) [258] | 6.49 (4.63-8.58) [136] | 6.07 (4.78-8.27) [218] | 6.65 (5.01-8.93) [157] | 0.29 |
| Neutrophils (G/L) | 4.75 (3.28-6.83) [768] | 4.64 (3.27-7.08) [258] | 4.91 (3.00-7.04) [135] | 4.54 (3.24-6.39) [218] | 5.11 (3.59-7.42) [157] | 0.20 |
| Lymphocytes (G/L) | 0.95 (0.64-1.31) [767] | 0.97 (0.63-1.32) [257] | 0.92 (0.63-1.32) [135] | 0.93 (0.64-1.27) [218] | 0.95 (0.66-1.32) [157] | 0.97 |
| CRP (mg/L) | 71.4 (30.0-135.1) [711] | 52.0 (20.6-103.9) [240] | 81.3 (42.8-140.3) [129] | 74.3 (29.9-138.1) [200] | 105.1 (37.1-160.1) [142] | <0.01 |
| Admission to ICU, n (%) | 181 (21.9) | 42 (15.1) | 34 (23.8) | 54 (23.0) | 51 (29.8) | <0.01 |
| *Admission directly to ICU* | 136 (16.4) | 22 (7.9) | 24 (16.8) | 45 (19.2) | 45 (26.3) | <0.01 |
| *Admission to general ward*  *and transfer to ICU during hospitalization* | 45 (5.4) | 20 (7.2) | 10 (7.0) | 9 (3.8) | 6 (3.5) | 0.39 |
| Death during hospitalization, n (%) | 170 (20.6) | 88 (31.7) | 28 (19.6) | 33 (14.0) | 21 (12.3) | <0.01 |
| NOTE : in square brackets [] : number of data available for the variable. If no square brackets, there is no missing data for the variable. | | | | |  |  |
| BMI: Body mass index, CRP : C-reactive protein, IQR : Interquartile range | | | | |  |  |
