# Supplementary material for: Baseline clinical features of COVID-19 patients, delay of hospital admission and clinical outcome: A complex relationship
Source: PLoS One. 2022 Jan 7;17(1):e0261428. doi: 10.1371/journal.pone.0261428 (PMC8741026; doi:10.1371/journal.pone.0261428)
Supplement: S2 Table — (DOCX) [file pone.0261428.s003.docx]

|  |  | **Patient age (years)** | | | |  |  |
| --- | --- | --- | --- | --- | --- | --- | --- |
| **Characteristics** | **All patients  n= 827** | **Patient age range [0-60[ n=200** | **Patient age range [60-70[ n=147** | **Patient age range [70-80[ n=187** | **Patient age range [80-90[ n=195** | **Patient age range [90-102] n=98** | **p-value** |
| Male gender, n (%) | 462 (55.9) | 130 (65.0) | 80 (54.4) | 123 (65.8) | 103 (52.8) | 26 (26.5) | <0.01 |
| BMI, median (IQR) | 26.2 (23.1-29.7) [664] | 28.0 (24.8-31.3) [161] | 27.1 (24.1-30.4) [118] | 26.2 (23.5-29.9) [152] | 24.5 (22.0-28.0) [151] | 23.5 (20.0-26.7) [82] | <0.01 |
| BMI ≥ 30, n (%) | 153 (23.0) [664] | 52 (32.3) [161] | 35 (29.7) [118] | 36 (23.7) [152] | 24 (15.9) [151] | 6 (7.3) [82] | <0.01 |
| Delay between symptom onset and hospital admission, median (IQR) |  | 7.0 (4.0-9.5) | 7.0 (4.0-10.0) | 6.0 (3.0-9.0) | 4.0 (2.0-7.0) | 3.0 (1.0-5.0) | <0.01 |
| Comorbidities, n (%) |  |  |  |  |  |  |  |
| *Cardiovascular disease* | 440 (53.2) | 46 (23.0) | 70 (47.6) | 114 (61.0) | 135 (69.2) | 75 (76.5) | <0.01 |
| *Diabetes* | 191 (23.1) | 29 (14.5) | 37 (25.2) | 55 (29.4) | 57 (29.2) | 13 (13.3) | <0.01 |
| *Malignancy* | 146 (17.7) | 8 (4.0) | 22 (15.0) | 34 (18.2) | 52 (26.7) | 30 (30.6) | <0.01 |
| *Chronic kidney disease* | 115 (13.9) | 13 (6.5) | 17 (11.6) | 32 (17.1) | 33 (16.9) | 20 (20.4) | <0.01 |
| *Chronic lung disease* | 103 (12.5) | 12 (6.0) | 21 (14.3) | 32 (17.1) | 24 (12.3) | 14 (14.3) | 0.02 |
| *Chronic liver disease* | 55 (7.5) [736] | 16 (11.6) [138] | 15 (11.5) [131] | 15 (8.4) [179] | 6 (3.1) [191] | 3 (3.1) [97] | <0.01 |
| *Immunodeficiency* | 48 (5.8) | 18 (9.0) | 7 (4.8) | 11 (5.9) | 9 (4.6) | 3 (3.1) | 0.21 |
| Smoking status, n (%) | [626] | [170] | [123] | [149] | [127] | [57] | <0.01 |
| *Current smoker* | 29 (4.6) | 8 (4.7) | 6 (4.9) | 12 (8.1) | 2 (1.6) | 1 (1.8) |  |
| *Ex-smoker* | 197 (31.5) | 31 (18.2) | 41 (33.3) | 68 (45.6) | 42 (33.1) | 15 (26.3) |  |
| *Never smoker* | 400 (63.9) | 131 (77.1) | 76 (61.8) | 69 (46.3) | 83 (65.4) | 41 (71.9) |  |

|  |  | **Patient age (years)** | | | |  |  |
| --- | --- | --- | --- | --- | --- | --- | --- |
| **Characteristics** | **All patients  n= 827** | **Patient age range [0-60[ n=200** | **Patient age range [60-70[ n=147** | **Patient age range [70-80[ n=187** | **Patient age range [80-90[ n=195** | **Patient age range [90-102] n=98** | **p-value** |
| Temperature at admission, median (IQR) | 38.0 (37.1-38.6) [739] | 38.2 (37.1-38.8) [174] | 38.0 (37.3-39.0) [132] | 38.0 (37.1-38.6) [166] | 38.0 (37.3-38.5) [180] | 37.5 (36.8-38.1) [87] | <0.01 |
| Symptoms at admission, n (%) |  |  |  |  |  |  |  |
| *History of fever/chills* | 674 (81.5) | 170 (85.0) | 126 (85.7) | 150 (80.2) | 154 (79.0) | 74 (75.5) | 0.15 |
| *Weakness* | 584 (70.6) | 138 (69.0) | 104 (70.8) | 124 (66.3) | 150 (76.9) | 68 (69.4) | 0.22 |
| *Cough* | 560 (67.7) | 156 (78.0) | 115 (78.2) | 126 (67.4) | 112 (57.4) | 51 (52.0) | <0.01 |
| *Shortness of breath* | 550 (66.5) | 121 (60.5) | 101 (68.7) | 134 (71.7) | 129 (66.2) | 65 (66.3) | 0.22 |
| *Diarrhea* | 229 (27.8) | 61 (30.5) | 38 (25.9) | 60 (32.1) | 50 (25.6) | 20 (20.4) | 0.21 |
| *Pain* | 225 (27.2) | 85 (42.5) | 62 (42.2) | 45 (24.1) | 24 (12.3) | 9 (9.2) | <0.01 |
| *Myalgia* | 144 (17.4) | 58 (29.0) | 37 (25.2) | 33 (17.7) | 12 (6.2) | 4 (4.1) | <0.01 |
| *Abdominal pain* | 62 (7.5) | 19 (9.5) | 20 (13.6) | 8 (4.3) | 11 (5.6) | 4 (4.1) | 0.05 |
| *Chest pain* | 50 (6.0) | 23 (11.5) | 9 (6.1) | 12 (6.4) | 4 (2.1) | 2 (2.0) | 0.38 |
| *Joint pain* | 12 (1.5) | 6 (3.0) | 3 (2.0) | 3 (1.6) | 0 (0.0) | 0 (0.0) | 0.64 |
| *Nausea* | 107 (12.9) | 34 (17.0) | 21 (14.3) | 17 (9.1) | 16 (8.2) | 19 (19.4) | 0.01 |
| *Headache* | 105 (12.7) | 54 (27.0) | 29 (19.7) | 15 (8.0) | 6 (3.1) | 1 (1.0) | <0.01 |
| *Confusion* | 93 (11.3) | 9 (4.5) | 10 (6.8) | 16 (8.6) | 37 (19.0) | 21 (21.4) | <0.01 |
| *Runny nose* | 72 (8.7) | 29 (14.5) | 17 (11.5) | 17 (9.1) | 7 (3.6) | 2 (2.0) | <0.01 |
| *Ageusia* | 64 (7.7) | 33 (16.5) | 11 (7.5) | 7 (3.7) | 6 (3.1) | 1 (1.0) | <0.01 |
| *Anosmia* | 58 (7.0) | 32 (16.0) | 14 (9.5) | 11 (5.9) | 6 (3.1) | 1 (1.0) | <0.01 |
| *Sore throat* | 34 (4.1) | 13 (6.5) | 5 (3.4) | 6 (3.2) | 5 (2.6) | 5 (5.1) | 0.30 |

|  |  | **Patient age (years)** | | | |  |  |
| --- | --- | --- | --- | --- | --- | --- | --- |
| **Characteristics** | **All patients  n= 827** | **Patient age range [0-60[ n=200** | **Patient age range [60-70[ n=147** | **Patient age range [70-80[ n=187** | **Patient age range [80-90[ n=195** | **Patient age range [90-102] n=98** | **p-value** |
| Biological parameters, median (IQR) |  |  |  |  |  |  |  |
| White blood cells (G/L) | 6.36 (4.87-8.59) [769] | 6.2 (4.7-8.0) [181] | 5.9 (4.4-8.4) [140] | 6.7 (5.3-8.9) [173] | 6.5 (5.0-9.1) [183] | 6.8 (5.1-8.8) [92] | 0.10 |
| Neutrophils (G/L) | 4.75 (3.28-6.83) [768] | 4.3 (3.0-6.1) [180] | 4.3 (2.9-6.5) [140] | 5.1 (3.8-7.3) [173] | 5.2 (3.3-7.3) [183] | 4.9 (3.5-7.3) [92] | <0.01 |
| Lymphocytes (G/L) | 0.95 (0.64-1.31) [767] | 1.1 (0.8-1.6) [180] | 1.0 (0.7-1.5) [140] | 0.9 (0.6-1.2) [173] | 0.8 (0.5-1.2) [182] | 0.9 (0.6-1.1) [92] | <0.01 |
| CRP (mg/L) | 71.4 (30.0-135.1) [711] | 56.9 (20.6-126.2) [161] | 65.7 (27.9-146.1) [128] | 82.6 (33.1-82.6) [157] | 91.9 (40.4-154.8) [173] | 59.8 (25.6-103.4) [92] | <0.01 |
| Admission to ICU, n (%) | 181 (21.9) | 52 (26.0) | 48 (32.7) | 52 (27.8) | 28 (14.4) | 1 (1.0) | <0.01 |
| *Admission directly to ICU* | 136 (16.4) | 44 (22.0) | 30 (20.4) | 40 (21.4) | 21 (10.8) | 1 (1.0) | <0.01 |
| *Admission to general ward and transfer to ICU during hospitalization* | 45 (5.4) | 8 (5.1) | 18 (15.4) | 12 (8.2) | 7 (4.0) | 0 (0.0) | <0.01 |
| Death during hospitalization, n (%) | 170 (20.6) | 11 (5.5) | 16 (10.9) | 39 (20.9) | 70 (35.9) | 34 (34.7) | <0.01 |
| NOTE : in square brackets [] : number of data available for the variable. If no square brackets, there is no missing data for the variable. | | | |  |  |  |  |
| BMI: Body mass index, CRP : C-reactive protein, IQR : Interquartile range | | |  |  |  |  |  |
