# Supplementary material for: Baseline clinical features of COVID-19 patients, delay of hospital admission and clinical outcome: A complex relationship
Source: PLoS One. 2022 Jan 7;17(1):e0261428. doi: 10.1371/journal.pone.0261428 (PMC8741026; doi:10.1371/journal.pone.0261428)
Supplement: Attachment — Submitted filename: Response to reviewers V3.docx [file pone.0261428.s006.docx]

November 16, 2021

**Answer letter**

**Reference : PONE-D-21-19254R1.**

Reviewer #3: The authors have addressed all of my previous concerns and clarified the details of the statistical analyses. I now also noticed that the sentence of line 78 "Our previous results showed that in COVID-19 patients, age as well as delay between symptom onset and hospital admission were both associated with ICU admission [11]" requires further clarifications. The authors should really include the number of patients in the cited study or state whether the cohort of reference [11] is the same as that of the submitted paper.

**R : Thank you for this comment. Our previous results were based on 417 patients. In the present work, we used the same cohort of reference, but with a larger number of patients. We have modified the Introduction section lines 70-71 and the Methods section line 84 accordingly.**
